# Robust production of heavy-chain-only antibodies in mice by CRISPR/Cas mediated *in situ* modification of *IgH* locus

**DOI:** 10.1093/nsr/nwag270

**Published:** 2026-05-12

**Authors:** Wei Wang, Shunan Zheng, Guanghai Xiang, Tao Feng, Yuanhao Peng, Yi Wu, Xiaoming Du, Pingxia Zhu, Yi Ru, Jing Zhang, Shuailin Wu, Haitang Han, Tian Huang, Gengsheng Cao, Haixue Zheng, Yaofeng Zhao, Haoyi Wang, Ran Zhang

**Affiliations:** State Key Laboratory of Animal Biotech Breeding, College of Biological Sciences, Frontiers Science Center for Molecular Design Breeding, National Engineering Laboratory for Animal Breeding, China Agricultural University, China; State Key Laboratory of Animal Biotech Breeding, College of Biological Sciences, Frontiers Science Center for Molecular Design Breeding, National Engineering Laboratory for Animal Breeding, China Agricultural University, China; State Key Laboratory of Immune Response and Immunotherapy, Institute of Health and Medicine, Hefei Comprehensive National Science Center, China; State Key Laboratory for Animal Disease Control and Prevention, College of Veterinary Medicine, Lanzhou University, Lanzhou Veterinary Research Institute, Chinese Academy of Agricultural Sciences, China; State Key Laboratory of Animal Biotech Breeding, College of Biological Sciences, Frontiers Science Center for Molecular Design Breeding, National Engineering Laboratory for Animal Breeding, China Agricultural University, China; Department of Laboratory Animal Sciences, School of Basic Medical Sciences, Capital Medical University, China; Rengene Biotechnology Co., Ltd., China; SAFE Pharmaceutical Services Corp, China; State Key Laboratory for Animal Disease Control and Prevention, College of Veterinary Medicine, Lanzhou University, Lanzhou Veterinary Research Institute, Chinese Academy of Agricultural Sciences, China; State Key Laboratory of Animal Biotech Breeding, College of Biological Sciences, Frontiers Science Center for Molecular Design Breeding, National Engineering Laboratory for Animal Breeding, China Agricultural University, China; State Key Laboratory of Animal Biotech Breeding, College of Biological Sciences, Frontiers Science Center for Molecular Design Breeding, National Engineering Laboratory for Animal Breeding, China Agricultural University, China; State Key Laboratory of Animal Biotech Breeding, College of Biological Sciences, Frontiers Science Center for Molecular Design Breeding, National Engineering Laboratory for Animal Breeding, China Agricultural University, China; School of Life Sciences, Henan University, China; School of Life Sciences, Henan University, China; State Key Laboratory for Animal Disease Control and Prevention, College of Veterinary Medicine, Lanzhou University, Lanzhou Veterinary Research Institute, Chinese Academy of Agricultural Sciences, China; State Key Laboratory of Animal Biotech Breeding, College of Biological Sciences, Frontiers Science Center for Molecular Design Breeding, National Engineering Laboratory for Animal Breeding, China Agricultural University, China; State Key Laboratory of Organ Regeneration and Reconstruction, Institute of Zoology, Chinese Academy of Sciences, China; University of Chinese Academy of Sciences, China; Beijing Institute for Stem Cell and Regenerative Medicine, China; State Key Laboratory of Animal Biotech Breeding, College of Biological Sciences, Frontiers Science Center for Molecular Design Breeding, National Engineering Laboratory for Animal Breeding, China Agricultural University, China

Heavy chain-only antibodies (hcAbs), initially identified in camelids, are composed exclusively of heavy chains [1]. Their variable regions, termed variable domain of heavy chain of heavy-chain antibodies (VHH) domains or nanobodies (Nbs), exhibit superior stability, compact size, and enhanced tissue penetration compared to conventional antibodies [2]. However, the reliance on camelids for Nb production presents significant limitations for broader application due to the practical challenges associated with maintaining and handling these animals compared to standard laboratory models. Recent advances have enabled the generation of hcAbs in mice and rats through the genomic integration of camelid or human heavy chain genes. Examples include cam/μMT mice [3], MGΔ, GΔ, and MΔGΔ mice [4], L-chain-deficient mice [5], LamaMice [6], and UniRats [7]. Notably, GΔ mice and UniRats, lacking the *μ* and *δ* genes critical for B-cell development, demonstrate that exogenous heavy chain gene expression can rescue B-cell developmental arrest caused by the absence of IgM and/or IgD. Nevertheless, current strategies have achieved only limited integration of variable gene segments into rodent genomes. The endogenous mouse *IgH* locus harbors a diverse repertoire of variable genes, which could theoretically support the generation of variable domain of heavy

chain (VHs) against a wide array of antigens. This premise previously motivated the development of HG1 mice and rats [8,9], engineered via *in situ* CH1 exon deletion from the *γ1/2a* genes. However, these modifications resulted in diminished *γ1/2a* expression and suboptimal immune responses. To overcome these limitations, this study proposes the development of an MDG1 mouse model using clustered regularly interspaced short palindromic repeats / CRISPR-associated protein 9 (CRISPR/Cas9)-mediated genome editing. The objective is to substantially enhance hcAbs’ expression while preserving normal B-cell development in the absence of *μ* and *δ* genes.

Firstly, we utilized CRISPR/Cas9 to precisely delete a 134 kb fragment spanning the genomic region from C*_μ_* to C*_γ2c-CH1_* at the heavy chain loci, generating MDG1 mice that exclusively express IgG2c (Fig. 1A and Fig. S1A). Animal care was conducted in accordance with the animal welfare guidelines of China Agricultural University. All animal experiments performed in this study were approved by the Animal Care and Use Committee of China Agricultural University. To investigate IgG2c hcAbs expression, we analysed *γ2c* gene transcription in the spleen compared to wild-type (WT) mice. Two shorter transcriptional splice variants of the *γ2c* gene were identified.

**Figure 1.**
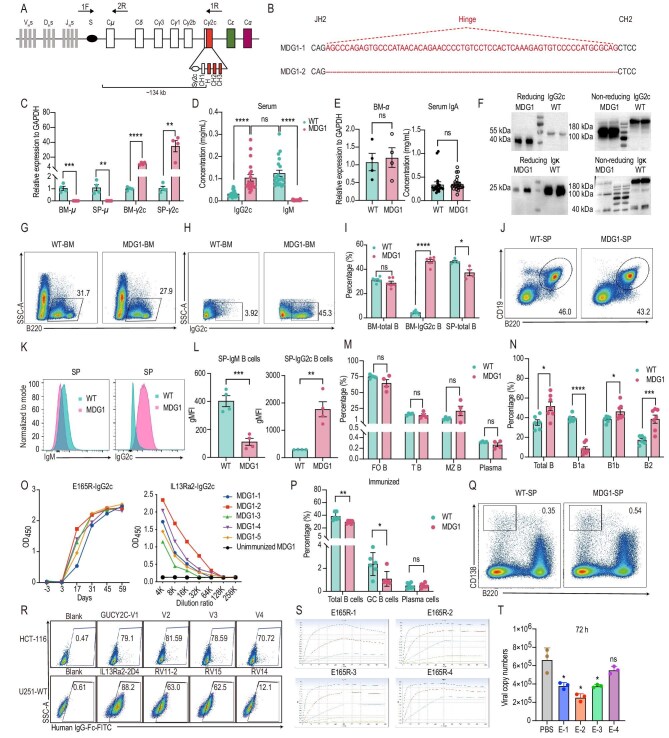
Characterization of B-cell development and immune functionality in MDG1 mice as a potential platform for sdAbs’ screening. (A) Construction strategy of MDG1 mice, with primers 1F, 1R, and 2R used for genotyping. (B) Sequence analysis of two transcriptional splice variants of the *γ2c* gene in MDG1 mice. (C) q-PCR analysis of the relative *γ2c* and *μ* gene expression in the BM and SP of MDG1 and WT mice (*n* = 4). (D) ELISA analysis of serum IgG2c and IgM levels in MDG1 and WT mice (*n* = 20). (E) q-PCR and ELISA analysis of BM *α* gene and serum IgA levels in MDG1 mice. (F) Western blot analysis of serum IgG2c and Igκ under both reducing and nonreducing conditions in MDG1 mice. Goat anti-mouse IgG2c-horseradish peroxidase (HRP) and Igκ-HRP antibodies were diluted at 1:5000. Flow cytometry analysis of BM B220^+^ (G) and B220^+^IgG2c^+^ (H) B cells in MDG1 and WT mice. (I) Quantitative histogram comparing the proportions of BM-B220^+^ total B cells, BM-IgG2c^+^, and SP-B220^+^CD19^+^ total B cells between MDG1 and WT mice. (J) Flow cytometry analysis of SP-B220^+^CD19^+^ in MDG1 and WT mice. (K) Flow cytometry analysis of splenic IgM^+^ B cells and IgG2c^+^ B cells in WT and MDG1 mice. (L) Histogram showing the comparison of gMFI of splenic IgM^+^ B cells and IgG2c^+^ B cells between WT and MDG1 mice. (M) Histogram showing the percentage of splenic FO B (B220^+^CD19^+^CD21^+^CD23^+^), T B (B220^+^CD19^+^CD21^−^CD23^−^), MZ B (B220^+^CD19^+^CD21^+^CD23^−^), and plasma cells (B220^low^CD138^hi^) in MDG1 and WT mice. (N) Histogram showing the percentage of peritoneal cavity CD19^+^ total B cells, B1a cells (CD19^+^CD23^−^CD5^+^), B1b cells (CD19^+^CD23^−^CD5^−^), and B2 cells (CD19^+^CD23^+^CD5^−^) in MDG1 and WT mice. (O) ELISA analysis of E165R- and IL13Ra2-specific IgG2c levels in the sera of MDG1 mice. Goat anti-mouse IgG2c-HRP antibody was diluted at 1:5000. (P) Histogram showing the percentage of B220^+^CD19^+^ total B cells, B220^+^CD19^+^Fas^+^CD38^low^ GC B cells, and B220^low^CD138^hi^ plasma cells in the spleens of MDG1 and WT mice immunized with E165R. (Q) Flow cytometry analysis of splenic B220^low^CD138^hi^ plasma cells in MDG1 and WT mice following E165R immunization. (R) Flow cytometry analysis of the binding capacity of GUCY2C-specific sdAbs to HCT-116 cells and IL13Ra2-specific sdAbs to U251-WT cells. The commercial anti-IL13Ra2 antibody (clone 2D4) was used as a positive control. The dosage of each sdAb used was 1 μg. (S) BLI assay of E165R-1, E165R-2, E165R-3, and E165R-4 sdAbs binding to Bio-E165R antigen immobilized on a biosensor. The sdAbs’ concentrations ranged from high to low: E165R-1 and E165R-4 at 5, 1.25, and 0.3 μg/ml; E165R-2 and E165R-3 at 5, 1.25, 0.3, 0.078, and 0.02 μg/ml. (T) q-PCR analysis of ASFV-GS/2018 viral copy number in PAM cells treated with E165R-1, E165R-2, E165R-3, and E165R-4 (abbreviated as E-1 to E-4) sdAbs after 72 h. The sdAbs dosage was 50 μg. Statistical analysis was performed using a two-tailed Student’s *t*-test. *P < 0.05, **P < 0.01, ***P < 0.001, ****P < 0.0001. SP, spleen; gMFI, geometric mean fluorescence intensity; ns, not significant.

The majority of the transcripts lacked only the CH1 exon, whereas a subset of them lacked both the CH1 and hinge exons (Fig. 1B and Fig. S1B). Quantitative real-time polymerase chain reaction (q-PCR) analysis showed significantly higher *γ2c* expression in the bone marrow (BM) and spleen of MDG1 mice, while the *μ* gene transcript was absent. Notably, the transcription level of *γ2c* even surpassed that of the *μ* gene in WT mice, which is responsible for initiating B-cell development (Fig. 1C and Fig. S1C). This could be attributed to the fact that MDG1 mice express only *γ2c* in the early B-cell development, thereby avoiding the competition between *μ* and *δ* genes in WT mice. Enzyme linked immunosorbent assay (ELISA) revealed a substantial increase in serum IgG2c levels in MDG1 mice compared to WT controls, reaching levels similar to those of IgM in WT mice. As expected, serum IgM was undetectable in MDG1 mice (Fig. 1D). The expression of the *α* gene did not obviously change, indicating an effective class-switch recombination from IgG2c to IgA (Fig. 1E and Fig. S1D). The abundance of serum Igκ was reduced in MDG1 mice compared with WT mice (Fig. S1E and F). Furthermore, Western blot analysis under reducing and nonreducing conditions confirmed exclusive IgG2c hcAbs expression in MDG1 mice, with no detectable IgM (Fig. 1F and Fig. S1G).

Taken together, we successfully generated MDG1 mice with high endogenous IgG2c hcAbs expression.

However, it is unclear whether hcAbs can support B cell receptor (BCR)-dependent development during early B-lineage cell maturation [10]. To investigate B-cell development dynamics in MDG1 mice, B cells from the BM were analysed by flow cytometry. No significant differences were observed between MDG1 and WT mice in either the percentage or total number of total B cells (B220^+^), pre B cells (B220^+^IgM^−^IgG2c^−^CD43^−^) and pro B cells (B220^+^IgM^−^IgG2c^−^CD43^+^CD24^+^) (Fig. 1G and I and Fig. S1H–K). As expected, IgM^+^ B cells were undetectable in MDG1 mice (Fig. S1L–N). In contrast, the proportion and number of IgG2c^+^ B cells were significantly increased, even exceeding the levels of IgM^+^ B cells in WT mice (Fig. 1H and I and Fig. S1M and N). Furthermore, flow cytometry analysis of spleen cells from MDG1 and WT mice revealed a slight reduction in the percentage of B220^+^CD19^+^ B cells in MDG1 mice, although the cell numbers were quite similar between the two groups (Fig. 1I and J and Fig. S1K). Notably, MDG1 mice exhibited higher expressing level of IgG2c^+^ B cells, compared to that of IgG2c^+^ B cells in WT mice. As expected, IgM^+^ B cells were absent in the spleens of MDG1 (Fig. 1K and L). Flow cytometry analysis of splenic B cells revealed that surface IgA⁺ B cells were relatively rare in the spleen, whereas κ light chain-expressing B cells were readily detectable in both WT and MDG1 mice (Fig. S1O). Moreover, IgG2c B-cell receptors in MDG1 splenocytes were co-expressed with κ light chains on the cell surface (Fig. S1P). Analysis of peripheral B-cell subsets revealed no significant differences in the proportions of follicular B (FO B), marginal zone B (MZ B), transitional B (T B), or plasma cells between MDG1 and WT mice, except for a slight reduction in plasma cells of MDG1 mice (Fig. 1M and Fig. S1Q and R). In the peritoneal cavity, MDG1 mice presented a higher percentage of total CD19^+^ B cells and a lower percentage of CD5^+^CD23^−^ B1a cells. However, the populations of CD5^−^CD23^−^ B1b and CD5^−^CD23^+^ B2 cells increased markedly (Fig. 1N and Fig. S1S and T), likely influenced by distinct BCR signaling pathways driven by the *μ* and *γ2c* genes in MDG1 and WT mice, respectively.

Considering that IgG2c hcAbs are the first expressed Ig in MDG1 mice, it is essential to explore their influence on Ig repertoires selection. We sequenced the Ig heavy chain variable region repertoire using spleen and BM cells from MDG1 mice and spleen cells from WT mice. *V_H_* and *J_H_* gene usage patterns, amino acid composition, and complementary determining region 3 (CDR3) length of IgG2c in MDG1 mice were largely similar to those of IgG2c and IgM in WT mice (Fig. S2A–D). Specifically, the CDR3 lengths of splenic IgG2c in MDG1 mice were predominantly 11 and 12 amino acids, whereas those in WT mice were mainly 9 or 10 amino acids in length. Interestingly, a significantly greater percentage of unique IgG2c heavy chain CDR3 was observed in MDG1 mice compared to WT mice (Table S1). Although IgG2c hcAbs cannot bind light chains, the usage frequencies of Igκ variable region *V_L_* genes and *J_L_* genes, along with CDR3 amino acid composition and length, remained consistent with those in WT mice, showing no discernible preference based on next generation sequencing (NGS) analysis (Fig. S2E–H).

MDG1 mice not only exhibited nearly normal B-cell development but also expressed high levels of IgG2c hcAbs, surpassing the performance of previous models, such as HG1 mice and HG1 rats. To evaluate their immune competence, MDG1 mice were immunized with a variety of antigens, including guanylate cyclase C (GUCY2C), interleukin 13 receptor alpha 2 (IL13Ra2), and dUTPase E165R from African swine fever virus (ASFV). ELISA results demonstrated that MDG1 mice produced substantial quantities of IgG2c-type hcAbs against all tested antigens (Fig. 1O and Fig. S3A). Flow cytometry analysis revealed significantly lower proportions of B220^+^CD19^+^ total B cells and B220^+^CD19^+^Fas^+^CD38^low^ germinal center (GC) B cells in MDG1 mice compared to WT controls, suggesting a potential impact of reduced immunoglobulin subtype diversity (Fig. 1P and Fig. S3B and C). However, the proportions and numbers of B220^low^CD138^hi^ plasma cells in MDG1 mice were comparable to those in WT mice, indicating effective antibody production following antigen stimulation (Fig. 1Q and Fig. S3D).

Following the successful induction of a robust immune response in MDG1 mice, we proceeded to screen antigen-specific single-domain antibodies (sdAbs) using phage display. Four rounds of screening were performed to isolate sdAbs clones specific to E165R, GUCY2C, and IL13Ra2, which were subsequently validated through ELISA. Interestingly, the GUCY2C- or IL13Ra2-specific sdAbs were derived from single clonotypes, respectively, and the same was observed for the E165R-specific sdAbs (Fig. S3E–G and Table S2). To evaluate their biophysical properties, the selected sdAbs were expressed and purified by affinity chromatography and size exclusion chromatography. A Kyte–Doolittle hydrophobicity plot confirmed the favorable hydrophilicity of these sdAbs (Fig. S4A and B). Moreover, these sdAbs exhibited long-term storage stability and thermostability comparable to those of camelid-derived VHH Nb mAb-5D9 (Fig. S4C and D). Binding specificity was assessed using a dot blot assay for E165R-specific sdAbs, showing a sensitivity of up to a picogram level (Fig. S5A). Furthermore, to evaluate the druggability of these sdAbs, GUCY2C- or IL13Ra2-specific clones were fused to a human IgG Fc domain, and the resulting hcAbs were analysed by flow cytometry. The results demonstrated that nearly all selected hcAbs targeted GUCY2C or IL13Ra2 effectively and selectively bound to their respective antigens on the surface of HCT-116, U251-WT, and 293T cells, indicating their potential utility in targeted immunotherapy. Notably, GUCY2C-specific hcAbs exhibited relatively lower fluorescence activated cell sorter (FACS) signals, which is likely due to the comparatively low surface expression of GUCY2C on these cells (Fig. 1R, Fig. S5B, and Table S3). Binding affinity was further evaluated using biolayer interferometry (BLI) with an Octet-RED system, where these sdAbs or hcAbs exhibited high binding affinities, ranging from 10^−8^ to 10^−10^ M (Fig. 1S, Fig. S5C, Table 1, and Table S4). We also assessed the virus-neutralizing capacity of E165R-specific sdAbs against ASFV. Notably, treatment of porcine primary alveolar macrophage (PAM) cells with sdAbs resulted in a significant reduction in virus copy numbers, highlighting their potential for virus detection and therapeutic applications (Fig. 1T and Fig. S5D).

**Table 1.** Affinity analysis of the screened sdAbs.

	*K*_D_ (M)	*k*_dis_ (1/s)	*k*_on_ (1/Ms)	Full *R*^2^
E165R-1	9.24 × 10^−9^	4.64 × 10^−5^	4.83 × 10^4^	0.9985
E165R-2	1.41 × 10^−10^	1.76 × 10^−5^	1.24 × 10^5^	0.9987
E165R-3	2.98 × 10^−10^	2.94 × 10^−5^	9.87 × 10^4^	0.9990
E165R-4	1.14 × 10^−9^	1.62 × 10^−4^	1.42 × 10^5^	0.9934

The dissociation constant [*K*_D_ (M)], on-rates [*k*_on_ (1/Ms)] and off-rates [*k*_dis_ (1/s)] for sdAbs binding to E165R.

In summary, we successfully generated the MDG1 mouse model expressing IgG2c hcAbs using CRISPR/Cas9. Remarkably, despite the absence of the *μ* and *δ* genes, MDG1 mice exhibited nearly normal B lymphocyte differentiation and development. Moreover, MDG1 mice demonstrated robust immune responses to various antigens, facilitating the screening of high-affinity and neutralizing sdAbs through phage display. These features position the MDG1 mouse model as a promising tool for sdAbs’ screening and B-cell development studying.

## Supplementary Material

nwag270_Supplemental_File
