# Supplementary material for: Robust production of heavy-chain-only antibodies in mice by CRISPR/Cas mediated in situ modification of IgH locus
Source: Natl Sci Rev. 2026 May 12;13(11):nwag270. doi: 10.1093/nsr/nwag270 (PMC13281104; doi:10.1093/nsr/nwag270)
Supplement: nwag270_Supplemental_File [file nwag270_supplemental_file.docx]

Supplementary Information for

**Robust production of heavy-chain-only antibodies in mice by CRISPR/Cas mediated *in situ* modification of IgH locus**

Wei Wang^1†^, Shunan Zheng^1†^, Guanghai Xiang^2†^, Tao Feng^3†^, Yuanhao Peng^1†^, Yi Wu^4^, Xiaoming Du^5^, Pingxia Zhu^6^, Yi Ru^3^, Jing Zhang^1^, Shuailin Wu^1^, Haitang Han^1^, Tian Huang^7^, Gengsheng Cao^7^, Haixue Zheng^3^, Yaofeng Zhao^1* ✉^, Haoyi Wang^8,9,10 *✉^ and Ran Zhang^1* ✉^

**^1^**State Key Laboratory of Animal Biotech Breeding, College of Biological Sciences, Frontiers Science Center for Molecular Design Breeding, National Engineering Laboratory for Animal Breeding, China Agricultural University, Beijing 100193, China.

**^2^**State Key Laboratory of Immune Response and Immunotherapy, Institute of Health and Medicine, Hefei Comprehensive National Science Center, Hefei 230601, China.

**^3^**State Key Laboratory for Animal Disease Control and Prevention, College of Veterinary Medicine, Lanzhou University, Lanzhou Veterinary Research Institute, Chinese Academy of Agricultural Sciences, Lanzhou 730000, China.

**^4^**Department of Laboratory Animal Sciences, School of Basic Medical Sciences, Capital Medical University, Beijing 100069, China.

**^5^**Rengene Biotechnology Co., Ltd., Beijing 100036, China.

**^6^**SAFE Pharmaceutical Services Corp, Beijing, China.

**^7^**School of Life Sciences, Henan University, Kaifeng 475004, China.

**^8^**State Key Laboratory of Organ Regeneration and Reconstruction, Institute of Zoology, Chinese Academy of Sciences, Beijing 100101, China

**^9^**University of Chinese Academy of Sciences, Beijing 100049, China.

**^10^**Beijing Institute for Stem Cell and Regenerative Medicine, Beijing 100101, China.

^†^These authors contributed equally.

*Corresponding author: Ran Zhang ([zhangran0628@cau.edu.cn](mailto:zhangran0628@cau.edu.cn)), Haoyi Wang ([wanghaoyi@ioz.ac.cn](mailto:wanghaoyi@ioz.ac.cn)) and Yaofeng Zhao ( [yaofengzhao@cau.edu.cn](mailto:yaofengzhao@cau.edu.cn)).

**The PDF file includes:**

Materials and Methods

Supplementary References

Author contributions, Acknowledgements and Conflict of interest disclosure

Supplementary Figures S1 to S6

Supplementary Tables S1 to S9

**Materials and Methods**

***Generation and genotyping of MDG1 mice***

Based on the immunoglobulin heavy chain complex data from C57BL/6J mice available on the NCBI website (Gene ID: 111507), we used CRISPR/Cas9 technology to precisely delete a 134 kb fragment extending from C*_μ_* to C*_γ2c-CH1_* of the immunoglobulin heavy chain loci, named MDG1 mice[1]. Initially, we designed four sgRNAs targeting the 5' end of the first exon of C*_μ_* and the 3' end of C*_γ2c-CH1_*. We then delivered the CRISPR/Cas9 components, including Cas9 mRNA, single-guide RNA, and donor oligonucleotide, into mouse zygotes via electroporation, achieving high-efficiency targeted mutations. Subsequently, we identified mouse genotypes using specifically designed amplification primers positioned upstream of the 5' end of the first exon of C*_μ_* and downstream of the 3' end of C*_γ2c-CH1_*. The sgRNA sequences and genotyping primers for genotyping MDG1 mice are listed in (**Table S5**). All WT mice were purchased from SPF (Beijing) Biotechnology Co., Ltd. Animal care was conducted in accordance with the animal welfare guidelines of China Agricultural University. All animal experiments performed in this study were approved by the Animal Care and Use Committee of China Agricultural University.

***RT-PCR* and *q-PCR***

For transcriptional analysis, total RNA was extracted from the spleen and BM of MDG1 or WT C57BL/6J mice using HiPure Total RNA Plus Micro Kit (R4121, Magen, China). The integrity, concentration and purity of RNA were analyzed via agar gel electrophoresis and Nanodrop 3000. Two micrograms of RNA were used for the reverse transcription reaction Using Abm All-In-One 5X RT kit (G490, Abm, Canada). RT-PCR detected the transcription of MDG1 IgG2c with specific primers. q-PCR was performed using Hieff qPCR SYBR Green Master Mix (11201ES08, Yeasen, China) with primers for *μ, γ2c*, *α* and *gapdh* under the following cycling conditions: 95°C for 15 min, followed by 45 cycles of 95°C for 10 s, 60°C for 20 s, and 72°C for 20 s. The relative transcription levels of *μ*, *γ2c* and *α* were calculated using the 2-^△△Ct^ method, comparing values with the internal control. The relative transcription level of MDG1 *γ2c* was also compared with the control WT *μ* in the same manner. Primers for RT-PCR and qPCR are listed in (**Table S6**).

***Western blot and ELISA analyses***

Serum samples were collected from 8-week-old MDG1 and WT C57BL/6J mice. The samples were separated by 8%-10% SDS-PAGE under either non-reducing conditions (without dithiothreitol (DTT)) or reducing conditions (with 5 mM DTT), and subsequently transferred onto Immobilon-P Transfer Membranes (Merck Millipore, IPVH00010). Immunodetection utilized HRP-conjugated antibodies, including goat anti-mouse IgM (*μ* chain) (Rockland Immunochemical, Limerick, PA, USA), goat anti-mouse IgG2c heavy chain (Abcam, Cambridge, MA, USA), goat anti-mouse IgA heavy chain (Abcam, Cambridge, MA, USA) and goat anti-mouse Igκ antibody (Jackson ImmunoResearch, PA, USA) Enhanced chemiluminescence and autoradiography were performed using Amersham ECL Prime Western Blotting Detection Reagent (GE, RPN2232) and Tanon 5200 Chemiluminescence imaging system. Serum samples from age-matched WT mice served as controls.

For Western blot analysis of serum IgA binding to Igκ in MDG1 and WT mice under non-reducing conditions, the PVDF membrane was first probed with an anti-IgA antibody. After detection, the membrane was stripped using stripping buffer and subsequently reprobed to detect Igκ expression under non-reducing conditions.

To assess the expression levels of Ig subtypes in MDG1 and WT mice by ELISA, plates were incubated with 2 μg/mL specific antigen overnight at 4 °C. After three washes with 0.05%TBST (50 mM Tris, 0.14 M NaCl with 0.05% tween20), plates were blocked with 1% BSA (dissolved in the same 0.05%TBST buffer). Diluted serum samples were then added to the wells and incubated for 1 hour at room temperature. After washing, dilluted HRP-conjugated secondary antibodies were added and incubated for 1 hour at room temperature, plates were washed as mentioned above and developed using TMB peroxidase substrate (Biolegend, San Diego, CA, USA), and absorbance was measured at 450 nm. The following HRP-conjugated secondary antibodies were used to detect specific Ig subtypes, all at a 1:5,000 dilution: IgG2c: Goat anti-mouse IgG2c-HRP (A90-136A, Bethyl, Montgomery, TX, USA), IgA: Goat anti-mouse IgA-HRP (A90-103P, Bethyl), IgM: Goat anti-mouse IgM-HRP (A90-101P, Bethyl).

For the ELISA used to evaluate antigen-specific antibody responses in MDG1 mice immunized with E165R, GUCY2C or IL13Ra2, the same procedure was followed. Goat anti-mouse IgG2c-HRP (A90-136A, Bethyl, Montgomery, TX, USA) was used as the secondary antibody at a 1:5,000 dilution.

***B cell flow cytometry***

B cells from the spleen, bone marrow and peritoneal cavity of MDG1 and WT C57BL/6J mice were harvested and prepared into cell suspensions. For all flow cytometry analysis staining, cells were incubated in buffer (PBS, 2% FBS) and stained with various conjugated antibodies at 4 °C for 20 minutes with various conjugated antibodies. The staining antibodies include APC-conjugated anti-mouse CD45R (B220) (No. RA3-6B2; eBioscience), PE-conjugated anti-mouse CD5 (No. ab114078; Abcam), APC-conjugated anti-mouse CD23 (No. ab25457; Abcam), BV421-conjugated anti-mouse CD138 (No. 562610; BD Pharmingen), PE/Cy7-conjugated anti-mouse CD95 (No. 553653; BD Pharmingen), APC/Cy7-conjugated anti-mouse CD38 (No. 102727; Biolegend), APC/Cy7-conjugated anti-mouse IgM (No. 406515; Biolegend), eFluor 450-conjugated anti-mouse B220 (No. RA3-6B2; eBioscience), eFluor 506-conjugated anti-mouse CD19 (No. 85-69-0193-80; eBioscience), PE-conjugated anti-mouse CD23 (No. 85-12-0232-82; eBioscience), PE/Cy7-conjugated anti-mouse CD21/CD35, CD19, IgM (No. 85-25-0211-80, 25-0193-82, 85-25-5890-82; eBioscience), Goat anti-Mouse IgG2c heavy chain (FITC) (ab97254, Abcam, UK), Goat anti-Mouse IgA alpha chain (FITC) (ab97234, Abcam, UK), BD Pharmingen^TM^ PE Rat anti-Mouse lgκ Antibody (562021, BD, USA), 7-AAD Viability Staining Solution (No. 85-00-6993-50; eBioscience).

To enhance the druggability properties and stability of GUCY2C- and IL13Ra2-specific sdAbs, the sdAbs were fused *in vitro* with the human IgG Fc domain. Binding specificity was then evaluated by flow cytometry using HCT-116 cells, U251-WT cells and 293T cells transfected to express the extracellular domains of GUCY2C or IL13Ra2. Goat anti-Human IgG Fc-FITC (2048-02, SouthernBiotech, USA) was used as the secondary antibody. Data were acquired using a BD flow cytometry analysis Canto flow cytometer with Flow cytometry analysis Diva software and analyzed using FlowJo.

***Library construction***

RNA was extracted from the spleens of MDG1 and WT mice, followed by reverse transcription as previously described. Spleen cDNAs from MDG1 mice were used for amplifying IgG2c and *κ* chain variable regions, while cDNAs from WT mice were used for amplifying the intact variable region of IgM, IgG2c and *κ* chain. The library for Next-generation sequencing (NGS) of variable segments from both MDG1 and WT mice was constructed using nested PCR reactions as described below:

In the first round PCR, the amplification conditions were described as follow: an initial denaturation step at 94°C for 3 min, followed by 30 PCR cycles of 94°C for 30 s, 50°C for 30 s, and 72°C for 1 min, and a final extension step of 72°C for 3 min. The primers were listed in (**Table S7-8**). MHV1-MHV12 served as upstream degenerate primers, IgG2C-CH2-R and IgM-CH2-R were the downstream primers for amplification of IgG2c and IgM variable regions, respectively; IGKV1-IGKV13 were upstream degenerate primers, and IGKC-R were downstream primers for the amplification of *κ* light chain variable region.

In the second round PCR, the amplification conditions were described as follows: an initial denaturation step at 94°C for 3 min, followed by 5 PCR cycles of 94°C for 30 s, 50°C for 30 s, and 72°C for 30 s, and followed by 20 PCR cycles of 94°C for 30 s, 50°C + 0.5 °C/per cycle for 30 s and 72°C for 30 s; followed by 5 PCR cycles of 94°C for 30 s, 60°C for 30 s, and 72°C for 30 s, a final extension step at 72°C for 5 min. Primers for the second round PCR were also listed in (**Table S7-8**). MHVF1-MHVF5 were used as upstream degenerate primers. WT-IG2C-CH1-R, KO-IG2C-CH2-R and WT-IgM-CH1-R served as downstream primer for the amplifying IgG2c and IgM variable regions in WT and MDG1 mice, respectively. IGKVF1-IGKVF9 and IGKC-R2 were used for amplifying the *κ* light chain variable region.

***Next-generation sequencing and data analysis***

To construct variable region sequence libraries, total bone marrow cells from the femurs and splenic cells were isolated from three WT and three MDG1 mice. cDNA libraries were subsequently generated from each cell population. Complete variable regions of IgG2c from bone marrow and spleen of MDG1 mice, as well as splenic IgM and IgG2c from WT mice, were amplified using nested PCR with the corresponding cDNA templates. For both MDG1 and WT mice, three technical repetitions were performed for each individual, and the samples from the three individuals were pooled together for sequencing. All samples were sent to Beijing Tangtang Tianxia Biotechnology Co., Ltd. for library quality inspection and sequencing. The second round of PCR products were purified using HiPure Gel Pure DNA Mini Kit (Magen, D2111-02) after agarose gel electrophoresis and then subjected to Illumina PE250 paired-end sequencing. Raw reads were initially processed using the Trimmomatic method for quality filtering, retaining bases with a quality score above 20. The filtered data were then screened by length, removing reads shorter than 50 bp or those with only one end. High-quality reads were aligned to reference V and J genes using the IMGT website (https://www.imgt.org/), with sequences selected based on alignment to the V gene for at least 96 bp and the J gene for at least 80%, with an e-value threshold of < 0.001 for subsequent analysis. Parameters analyzed include frequency analysis of V gene and J gene usage, Length and amino acid composition of the CDR3, N/P nucleotide length, somatic hypermutation frequency.

***Immunization and ELISA detection of antigen specific immune response***

MDG1 mice and WT mice aged 6-7 weeks were immunized four or five times with African Swine Fever virus antigen E165R (donated by Lanzhou Veterinary Research Institute), GUCY2C (GUC-C52H6, ACRO Biosystems, China) or IL13Ra2 (10350-H08H, Sino Biological, China). The primary immunization was administered via subcutaneous injection using Freund’s complete adjuvant (FCA) (Sigma-Aldrich, St. Louis, MO, USA). Booster immunizations were performed every two weeks, primarily via intraperitoneal injection with Freund’s incomplete adjuvant (FIA) (Sigma-Aldrich, St. Louis, MO, USA), at a dosage of 100 μg per antigen per injection. To assess the immune response by ELISA, serum of MDG1 immunized with E165R was collected before the first immunization and three days after each booster, diluted 8000 times, and added to antigen-coated and skim milk-blocked plates. Serum of MDG1 immunized with GUCY2C and IL13Ra2 was collected after the last booster, diluted 4000, 8000, 16000, 32000, 64000,128000 and 256000 times, and added to antigen-coated and skim milk-blocked plates. The following procedure was according to the manufacturer’s instructions (Bethyl, Montgomery, TX, USA). All animal procedures were approved by the Animal Welfare Committee of China Agricultural University (Approval No. AW03111202-3-1).

***Phage display library construction***

The VHH gene library for Phage display screening from immunized MDG1 mice was amplified using nested PCR as described below. The amplification conditions and primers for IgG2c VHH in the first round PCR were identical to those used in the first round PCR for the construction of the NGS library. The primers for the second round PCR were listed in (**Table S9**). The MHVF (1-5)-*sfi* I were the five upstream degenerate primers and IgG2C-CH2-*sfi* I were the downstream primers used to amplify the IgG2c variable region of MDG1 mice. All primers were modified with a different *sfi* I site to facilitate cloning of the target sequence into the display vector. The VHH segments, once digested by *sfi* I (NEB, Ipswich, MA, USA), were cloned into the phage display vector pComb3XSS using T4 ligase (NEB, Ipswich, MA, USA) to construct the VHH plasmids library. These plasmids were then transformed into *E. coli* strain ER2738 by electroporation to generate VHH libraries, which were cultured at 37°C on 2×YT medium containing 100 μg/mL ampicillin, 100 μg/mL tetracycline and 1 M glucose. The VHH phagemid libraries were rescued with M13 helper phages (NEB, Ipswich, MA, USA) and precipitated with PEG8000-NaCl (20% PEG8000 and 0.5 M NaCl), and resuspended in PBS at last.

***Phage display panning and phage ELISA***

Antigens were coated on the plates overnight at concentrations (10 μg/mL, 5 μg/mL and 2.5 μg/mL) for every panning round at 4°C. Wells were washed with 0.1% PBST three times and blocked with 2% skim milk in 0.1% PBST at 25°C for 2 hours. After three additional washes with 0.1% PBST, about 1×10^10^ pfu/100 μL of phage particles in PBS were added to the wells and incubated at room temperature for an hour. Unbound phage particles were removed by washing ten times with 0.1% PBST, and antigen-specific phage particles which were assumed to be bound to the antigens coated on the wells were collected by elution with 100 μL of Gly-HCl (pH=2.2) for the first and second rounds or 5 μg/mL antigen for the remaining rounds on a room shaker for an hour. Selected phage clones were then used to infect *E. coli* strain ER2738 and were precipitated with PEG8000–NaCl (20% PEG and 0.5 M NaCl) for subsequently rounds of screening.

Phage ELISA was used to measure the binding property of antigen-specific phage clones, which were obtained after 4 rounds of panning. The plates were coated with 2 μg/mL antigen (~100 μL per well) and blocked with 3% skim milk in 0.1% PBST at 25°C for 2 hours. Then, 50 μL of phage clones and 0.1 μg of antigen were added into contention wells, while 50 μL of phage clones and 50 μL PBS were added into sample wells. The plates were incubated at room temperature for an hour, washed five times with 0.1% PBST, and subsequently incubated with an HRP-conjugated anti-M13 monoclonal antibody (Abcam, Cambridge, MA, USA). After five washes with 0.1% PBST, the plates were incubated with the TMB peroxidase substrate (Biolegend, San Diego, CA, USA). Absorbance was then measured at 450 nm.

For the GUCY2C or IL13Ra2 binding ELISA, the screened sdAbs dosage was 1 μg/well and the dilution of HRP-goat-anti-human Fc was 1:4000. The 5F9 and 1602 antibodies targeting GUCY2C was positive control[2], and the 2D4 antibody targeting IL13Ra2 was also positive control.

***Prokaryotic Expression and Purification***

Plasmids DNA was transformed into *E. coli* strain BL-21 for protein expression, extracted from bacterial clones containing antigen-specific VHH fragments. The bacterial clones were grown on the LB medium and stimulated with 0.1 M IPTG at 30 °C or 37°C overnight to induce the production of the sdAbs. The sdAbs were purified using Ni–NTA His Bind resin (No. 70666, Merck, Germany) following the manufacturer’s protocol. The purified sdAbs were dialyzed with PBS. The sdAbs were further purified by size exclusion chromatography (SEC) on a Superdex 75 pg Hiload 16/600 column (Cytiva) with running buffer PBS, pH 7.5 according to the manufacturer’s instructions.

***Biofilm layer interference and Dot blot***

The affinity of purified sdAbs was detected by Fortebio Octet RED96 with antigens were labeled with EZ-link Sulfo-NHS-LC-biotin (No. 21335, Thermo, USA) and were Desalted by Zeba™ Spin Desalting Columns (No. 89882, Thermo, USA), following the manufacturer’s protocol. The sdAbs were diluted in assay buffer (0.02% PBST supplemented with 0.1% BSA). The Octet program setting were as follows: Baseline 1 (180 s) - Loading (600 s) - Baseline 2 (120 s) - Binding (Association) (500 s) - Disassociation (500 s), the measured temperature was set at 30°C and the sample plate speed at 1,000 rpm. Data analysis was conducted using Fortebio analysis software with the last 10 s of baseline 2 as the starting point.

For the Dot blot, antigens to be tested were serially diluted by PBS and spotted onto PVDF membranes. After blocking the PVDF with 5% non-fat milk, the diluted sdAbs (500 ng/mL) were incubated with antigen-coated PVDF at room temperature for 2 hours. The membranes were washed five times with TBST and incubated with an HRP-conjugated anti-HA antibody (1:5,000) (003-101-005, AlpVHHs, China). Detection was performed using Amersham ECL Prime Western Blotting Detection Reagent (GE, RPN2232) and Tanon 5200 Chemiluminescence imaging system after five washes with TBST.

***Neutralization experiments with African swine fever virus***

We mixed four E165R-specific sdAbs or PBS with ASFV-GS/2018 strains, respectively. The dosage of sdAbs was 50 μg and the titer of virus was 1×10^6.52^ HAD50/mL. The mixture was co-incubated at room temperature for 1 h, then added to the porcine PAM cells and incubated at room temperature for 1 h, followed by replacement with normal 10% Fetal Bovine Serum (FBS) DMEM medium. Cells were collected 24h, 48h and 72 h, and total RNA was extracted for q-PCR to detect the copy number of ASFVs.

***Nanoscale Differentiation Scanning Fluorimetry***

Prometheus NT.48 instrument (NanoTemper Technologies) was used to measure the intrinsic tryptophan fluorescence changes of ET516-treated sdAbs with an excitation wave length of 280 nm. A 20 μL sample of sdAbs (0.2 mg/mL) was incubated with a series concentration of ET516 were loaded with capillaries and the heating process increased from 25 to 81.5 ℃ at a ramp rate of 1.5 ℃/min, Fluorescence quenching data were from the fluorescence scan step. The ratio of emission intensities (Em350nm/Em330nm) was recorded and plotted as a function of temperature. The fluorescence intensity ratio and first derivative were analyzed from PR, Therm Control and further processed with GraphPad Prism 10.

***Long-term storage stability***

The selected sdAbs were incubated at 4℃, 37℃, or 45℃ for 1, 3 and 7 days. Antigen-binding affinity was subsequently evaluated by ELISA. The VHH nanobody mAb-5D9, specific to Atrazine and isolated from camel by phage display, was used as a positive control[3]. To assess its binding activity, ovalbumin-conjugated Atrazine was used as the coating antigen. Both the sdAbs and coated antigen were used at a concentration of 2 μg/mL. Detection was performed using an HRP-conjugated anti-HA antibody (1:5,000 dilution; 003-101-005, Alpvhhs, China).

***Kyte-Doolittle Plot***

The amino acid sequences of sdAbs were input into the ProtScale tool of ExPasy, using the Kyte-Doolittle hydrophobicity scale option selected. The window size was set to 9 and a linear weight variation model was utilized [4, 5]. The results were output in a minimal numerical format to facilitate plot visualization using GraphPad Prism 10 software. Whole protein composition analysis of the sdAbs was performed using Protean software.

***Statistical analysis***

Statistical analyses were conducted using GraphPad Prism 10 software (GraphPad Software). Each experiment was repeated at least three times or included more than three individuals. The results are presented as means ± SEM. Differences between two groups were discerned using the two-tailed Student's *t*-test and indicated in the text as follow *P< 0.05, ** P< 0.01, *** P< 0.001, **** P< 0.0001, ns, no significant.

***Data and materials availability:***

The raw sequence data of NGS reported in this paper are publicly available at https://www.ncbi.nlm.nih.gov/sra with the accession number: PRJNA1194360.

**Supplementary References**

1.	Qin WN, Dion SL, Kutny PM *et al.* Efficient CRISPR/Cas9-Mediated Genome Editing in Mice by Zygote Electroporation of Nuclease. *Genetics*. 2015; **200**: 423-30.

2.	Rampuria P, Mosyak L, Root AR *et al.* Molecular insights into recognition of GUCY2C by T-cell engaging bispecific antibody anti-GUCY2CxCD3. *Sci Rep*. 2023; **13**: 13408.

3.	He QQ, Wang M, Zhao YJ *et al.* Isolation of atrazine nanobodies enhanced by depletion of anti-carrie protein phages and performance comparison between the nanobody and monoclonal antibody derived from the same immunogen. *Analytica Chimica Acta*. 2023; **1244**: 340848.

4.	Eisenberg D, Schwarz E, Komaromy M *et al.* Analysis of membrane and surface protein sequences with the hydrophobic moment plot. *J Mol Biol*. 1984; **179**: 125-42.

5.	Gasteiger E, Gattiker A, Hoogland C *et al.* ExPASy: the proteomics server for in-depth protein knowledge and analysis. *Nucleic Acids Res*. 2003; **31**: 3784-88.

6.	Li SF, Jiang KP, Wang T *et al.* Nanobody against PDL1. *Biotechnol Lett*. 2020; **42**: 727-36.

7.	Hong J, Kwon HJ, Cachau R *et al.* Dromedary camel nanobodies broadly neutralize SARS-CoV-2 variants. *Proc Natl Acad Sci U S A*. 2022; **119**: e2201433119.

**Funding statement:**

This work was supported by National Key Research and Development Program of China (2021YFA0805905, 2022YFD1300704 and 2023YFF1000900 to Y.Z.) and 2115 Talent Development Program of China Agricultural University (to R.Z.).

**Author Contributions**

Y.F.Z., H.Y.W., R.Z., and H.X.Z. planned the project and experimental design. G.H.X., H.Y.W., Y.W., X.M.D., and P.X.Z. constructed the MDG1 mouse. W.W., S.N.Z., G.H.X., Y.H.P., J.Z., S.L.W., T.F., and Y.R. performed and analyzed the experiments. W.W., S.N.Z., Y.H.P., R.Z. interpreted the results. S.N.Z., T.H., and G.S.C. processed and analyzed the NGS data. W.W., S.N.Z., and R.Z. wrote the manuscript. Y.F.Z., H.Y.W., R.Z., H.X.Z., and G.H.X. reviewed the manuscript.

**Conflict of interest disclosure:**

A patent application (H.Y.W., G.H.X., X.M.D. and Y.W. as inventors) has been filed by Rengene Biotechnology Co., Ltd., H.Y.W. has shares in Rengene Biotechnology Co., Ltd., which part funded the research grant for this project. The remaining authors declare no competing interests.

**Supplementary figure legends**

**Supplementary Fig. 1**

**Supplementary Fig. 1.** **Expression of immunoglobulins and B cell development in MDG1 mice**.

**(A)** The fragment size with primers 1F and 1R was approximately 685 bp in MDG1 mice, compared to 730 bp in WT mice using primers 1F and 2R. **(B)** RT-PCR and sequence analysis of *γ2c* gene transcription in MDG1 and WT mice. Upstream primers were positioned in the J_H_2 gene and downstream primers in the C_H_2 exon, generating amplicons of approximately 530 bp in MDG1 and 825 bp in WT mice. Two *γ2c* transcription forms are shown on the right. **(C)** Comparison of *γ2c* gene expression in bone marrow (BM) and spleen of MDG1 mice with *μ* gene expression in WT mice (n=4). **(D)** Western blot analysis of serum IgA levels in MDG1 and WT mice was performed under both reducing and non-reducing conditions (left panel). Western blot analysis of serum IgA binding to Igκ in MDG1 and WT mice under non-reducing conditions (right panel). The goat-anti-mouse Igκ-HRP and goat-anti-mouse IgA-HRP antibodies were diluted at 1:5,000. **(E-F)** Western blot analysis of serum Igκ levels in MDG1 and WT mice under reducing conditions (n=5). Equal volume of serum (1 μL) was loaded and the integrated density of the bands was quantified using Image J. The goat-anti-mouse Igκ-HRP was diluted at 1:5,000. **(G)** Western blot analysis of serum IgM levels in MDG1 mice under reducing conditions. The goat anti-mouse IgM-HRP was diluted at 1:5,000. **(H)** Flow cytometry analysis of BM pre B cells (B220^+^IgM^-^IgG2c^-^CD43^-^) and pro B cells (B220^+^IgM^-^IgG2c^-^CD43^+^CD24^+^) in MDG1 and WT mice. **(I-J)** The proportions **(I)** and cell numbers **(J)** of each B cell subset were shown in histograms. **(K)** Histogram depicting the cell numbers of BM B220^+^ total B cells and spleen B220^+^CD19^+^ total B cells from MDG1 and WT mice. **(L)** Flow cytometry analysis of B220^+^IgM^+^ BM B cells in MDG1 mice. **(M)** Comparison of the percentages of BM B220^+^IgM^+^ B cells in WT and B220^+^IgG2c^+^ B cells in MDG1 mice. **(N)** Histogram showing the cell numbers of BM B220^+^IgM^+^ B cells in WT and B220^+^IgG2c^+^ B cells in MDG1 mice. **(O)** Flow cytometry analysis of splenic IgA^+^ and Igκ^+^ B cells in MDG1 and WT mice. Histogram showing the percentage and cell numbers of IgA^+^ and Igκ^+^ B cells in WT and MDG1 mice. **(P)** Flow cytometry analysis of splenic IgG2c^+^Igκ^+^ B cells in WT and MDG1 mice. The gating of IgG2c^+^ cells was based on the IgG2c FMO control in WT mice. Histogram showed the percentage of splenic IgG2c^+^Igκ^+^ B cells in WT and MDG1 mice (n=4). **(Q)** Flow cytometry analysis of splenic follicular B cells (FOB: B220^+^CD19^+^CD21^+^CD23^+^), transitional B cells (TB: B220^+^CD19^+^CD21^-^CD23^-^), and marginal zone B cells (MZB: B220^+^CD19^+^CD21^+^CD23^-^) in MDG1 and WT mice. Histograms depict the cell numbers of each B cell subset. **(R)** Flow cytometry analysis of splenic plasma cells (B220^low^CD138^hi^) in MDG1 and WT mice, with histograms depicting their cell numbers. **(S-T)** Flow cytometry analysis of CD19^+^ total B cells **(S)**, B1a (CD19^+^CD23^-^CD5^+^), B1b (CD19^+^CD23^-^CD5^-^), and B2 (CD19^+^CD23^+^CD5^-^) cells **(T)** in the peritoneal cavity of MDG1 and WT mice. Statistical analysis was performed using a two-tailed Student's *t-test.* *P< 0.05, **P< 0.01, ***P< 0.001, ****P< 0.0001, ns, not significant.

**Supplementary Fig. 2**

**Supplementary Fig. 2.** **Characterization of the variable region in MDG1 mice by NGS.**

**(A-B)** Comparison of V_H_ family and J_H_ gene usage percentages between 8-week-old MDG1 and WT mice. **(C)** Comparison of heavy chain CDR3 amino acid usage patterns between MDG1 and WT mice. **(D)** Length distribution analysis of unique heavy chain CDR3 regions in MDG1 and WT mice. **(E-F)** Frequency analysis of V_L_ family and J_L_ gene family utilization in MDG1 mice. **(G)** Analysis of light chain CDR3 amino acid composition in MDG1 mice. **(H)** Length distribution analysis of unique light chain CDR3 regions in MDG1 mice. The number of mice in each of the MDG1 and WT groups was three.

**Supplementary Fig. 3**

**Supplementary Fig. 3**. **Identification and screening of antigen-specific sdAbs post-immunization.**

**(A)** ELISA analysis of GUCY2C- specific IgG2c levels in the sera of MDG1 mice. Goat anti-mouse IgG2c-HRP antibody was diluted at 1:5,000. **(B-C)** Flow cytometry analysis of splenic B220^+^CD19^+^ total B cells **(B)** B220^+^CD19^+^Fas^+^CD38^low^ GC B cells **(C)** in MDG1 mice immunized with E165R. Histogram depicts the number of total B cells and GC B cells. **(D)** Histogram showing the number of B220^low^CD138^hi^ plasma cells. **(E)** Phage ELISA analysis of clones capable of binding to the antigen E165R. The coated antigen concentration was 2 μg/mL. **(F)** Amino acid sequences of the sdAbs E165R-1, E165R-2, E165R-3, and E165R-4. **(G)** ELISA analysis of GUCY2C and IL13Ra2 binding by screened sdAbs, using 1608, 5F9-IgG1, 2D4, YM-mIg, and YM-scFv as positive controls, and PBS as a negative control. The sdAb dosage was 1 μg/well, and the HRP-conjugated goat anti-human Fc antibody was diluted at 1:4,000. Statistical analysis was performed using a two-tailed Student's *t-test*. *P< 0.05, ns, not significant.

**Supplementary Fig. 4**

**Supplementary Fig. 4. Hydrophobicity and stability analysis of antigen-specific sdAbs.**

**(A)** SEC purification profiles of E165R- and IL13Ra2-specific sdAbs using PBS (pH 7.5) as the elution buffer. The mAb-5D9, an Atrazine-specific VHH nanobody isolated from camel via phage display, was used as a control. **(B)** Kyte-Doolittle hydrophobicity plot of all screened sdAbs, as well as PDL1-VHH1 [6] and SARS-CoV-S1-1B5 sdAbs [7] with CDR1, CDR2, and CDR3 regions shaded in grey. The window size is set to 9, and the y-axis represents the hydrophobicity scores of the sdAbs. **(C)** Long-term storage stability analysis of the sdAbs under 4℃, 37℃ and 45℃ for 1, 4, and 7 days. Antigen-binding capacity of sdAbs was assessed by ELISA. The concentration of sdAbs and coated antigen was 2 μg/mL. mAb-5D9 served as a control. **(D)** Thermostability of sdAbs analyzed by Differential Scanning Fluorimetry (DSF). The plot showed the melting temperature (Tm) of E165R- and IL13Ra2-specific sdAbs, and mAb-5D9 was served as a control. DSF was performed with 20 μL samples of sdAbs at 0.2 mg/mL.

**Supplementary Fig. 5**

**Supplementary Fig. 5.** **Characterization of the affinity and activity of antigen-specific sdAbs and hcAbs.**

(**A**) Dot blot analysis of expressed E165R specific sdAbs, with irrelevant antigens P34, P54 and OVA as negative controls. All sdAbs were used at 500 ng/mL, and detection was performed using a 5,000-fold diluted mouse anti-HA-HRP antibody. (**B**) Flow cytometry analysis of the binding ability of GUCY2C- or IL13Ra2-specific hcAbs to 293T cells expressing GUCY2C or IL13Ra2, as well as to wild-type 293T cells. The antibodies 5F9 and 1608 targeting GUCY2C and 2D4 targeting IL13Ra2 served as positive controls. The dosage of hcAbs was 1 μg and the secondary antibody was goat anti-human IgG Fc-FITC (1:5000 dilution). (**C**) BLI analysis of the binding kinetics of GUCY2C- or IL13Ra2-specific monovalent sdAbs or bivalent hcAbs. GUCY2C or IL13Ra2 monovalent sdAbs were tested at the following concentrations (nM): 1500, 500, 250, 125, 61 and 10. GUCY2C-hcAbs were tested at the following concentrations (nM): 3000, 1000, 333.3, 111.1 and 37.03. IL13Ra2-hcAbs were tested at 1500, 750, 350, 187.5 nM. The concentration of GUCY2C and IL13Ra2 protein used in BLI analysis was 0.5 mg/mL each. (**D**) q-PCR analysis of ASFV-GS/2018 viral copy numbers in primary alveolar macrophage cells treated with E165R-1, E165R-2, E165R-3, and E165R-4 sdAbs for 24 and 48 h. The dosage of sdAbs was 50 μg. Statistical analysis was performed using a two-tailed Student's *t-test*. *P< 0.05, **P< 0.01, ns, not significant.

**Supplementary Fig. 6**

**Supplementary Fig. 6.** **Full Western Blots of all cropped images.**

(**A**) Original Western bot images of serum IgG2c under non-reducing and reducing conditions (Fig. 1F). (**B**) Original Western blot images of serum Igκ under non-reducing and reducing conditions (Fig. 1F). (**C**) Original Western blot images of serum IgA and Igκ under non-reducing and reducing conditions (Fig. S1D). (**D**) Original Western blot images of serum Igκ under non-reducing and reducing conditions (Fig. S1E). (**E**) Original Western blot images of serum IgM under reducing conditions (Fig. S1G).

**Supplementary tables**

**Supplementary Table 1. Number of raw and unique reads in the four Ig-subtype libraries.**

| **Sample** | **Raw reads** | **Unique reads** | **Unique reads/raw reads** |
| --- | --- | --- | --- |
| WT-SP-IgM | 1,365,745 | 277,057 | 20.29% |
| WT-SP-IgG2c | 2,292,397 | 13,351 | 0.58% |
| MDG1-BM-IgG2c | 669,155 | 79,063 | 11.82% |
| MDG1-SP-IgG2c | 1,243,365 | 111,964 | 9.00% |

The sequencing reads of WT IgG2c, IgM, and MDG1 IgG2c that aligned to the variable region were classified as raw reads. Among these, reads with a distinct CDR3 composition were identified as unique reads. The ratio between raw and unique reads reflects the diversity of the Ig repertoire.

**Supplementary** **Table 2.** **Amino acid sequence of sdAbs.**

| **Name** | **Amino acid sequence** |
| --- | --- |
| E165R-1 | EVKLLESGGGLVQPGGSLKLSCAASGFDFSRYWMSWVRQTPGKGLEWIGEINPESSTINYTPSLKDKFIISRDNAKNTLYLQMSKVRSEDTALYYCTRGTMITTDLAFWGQGTLVTVSSEPRVPITQNPCPPLKECPPCAAPDLLGGGQAGQ |
| E165R -2 | EVQLQQSGAELAKPGASVKVSCKASGYTFTSHWITWVRLRPGQALEWIGHIYPGSDTTNYSEKFKNKATLTVDTSSSTAYMQLTSLTSEDSAVYYCARGKFRHNNPYYFDYWGQGTTLTVSSEPRVPITQNPCPPLKECPPCAAPDLLGGGQAGQ |
| E165R -3 | EVQLQQSGAELVKPGASVKMSCKTSGYTFTTYWITWVRLRPGQALEWIGEIYPGSDKTNYSEKFKNKATLTVDTSSSTAYMQLSSLTSGDSAVYYCARGKVRYNNPYYFDYWGQGTTLTVSSEPRVPITQNPCPPLKECPPCAAPDLLGGGQAGQ |
| E165R -4 | QVQLQQSGAELVKPGASVKMSCKTSGYTFTTYWITWVRLRPGQALEWIGEIFPGNGNTNYNEKFKNKATLNVDTSSNTAYMQLNSLTSGDSAVYYCARGKFRYNNPYYFDDWGQGTTLTVSS-----------------------------APDLLGGGQAGQ |
| GUCY2C-V1 | EVQLQQSGPELVKPGASVKMSCKASGYTFTDYNMHWVKQSHGKSLEWIGYINPNNNNTAYNQKFKGKARLTVNKTSSTAYMELRSLTSEDSAVYYCARHYYGSSHMDYWGQGTSVTVSS |
| GUCY2C-V2 | EVQLQQSGPELVKPGASVKMSCKASGYTFTDYNMHWVKQSHGKSLEWIGYINPNTNNTAYNQKFKGKARLTVNKTSSTAYMELRSLTSEDSAVYYCARHYYGSSHMDYWGQGTSVTVSS |
| GUCY2C-V3 | EVQLQQSGPELVKPGASVKMSCKASGYTFTDYNMHWVKQSHGKSLEWIGYINPNNNNTAYNQKFKGKARLTVNKTSSTAYMELRSLTSEDSAVYYCANRHYYGTSHMDYWGQGTSVTVSS |
| GUCY2C-V4 | EVQLQQSGPELVKPGASVKMSCKASGYTFTDYNMHWVKQSHGKSLEWIGYINPNNNNTAYNQKFKGKARLTVNKTSSTAYMELRSLTSEDSAVYYCANRHYYGTNHMDYWGQGTSVTVSS |
| IL13Ra2-RV11-2 | EVKLVESGGGLVQPGGSMKLSCVASGFTFSNYWMNWVRQSPGKGLQWVAQIRLKSDNYATHYAESVKGRFTISRDDSKSSVYLQMNDLRTDDTGIYHCTYGKHDYWGQGTTLTVSS |
| IL13Ra2-RV14 | EVKLVESGGGLVQPGGSMKLSCVTSGFTFRNNWMNWVRQSPEKGLEWVAQIRLRSDNYATHYAESVKGRFTISRDDSKSSVYLQMNNLRAEDTGIYYCTVYGKPDYWGQGTTLTVSS |
| IL13Ra2-RV15 | EVKLVESGGGLVQPGGSMKLSCLASGFTFSSYWMNWVRQSPGKGLQWVAQIRLKSDNYATHYAESVEGRFTISRDDSRSSVYLQMNNLRPEDTGIYYCTVGEPDYWGQGTTLTVSS |
| PDL1-VHH1 | QVQLVESGGGSVQAGGALRLSCAASGYTSTSNCMGWFRQAPGKEREGVAAIYTGGGSTYYADSVKGRFTISQDNAKNTVYLQMNSLKPEDTAMYYCAAGDLDPAEYSDYDPTVFNYWGQGTLVTVSS |
| SARS-CoV-S1-1B5 | DVOLVESGGGSVOAGGSIRISCTGSRYTYSTYCMGWFROAPGKEEEAVAIINSGGGEPYYGDSVKGREFTISODRAKNTVYLOMDGLOPDDTAIYYCVAADSHNSRCYIGRSYVNYWGOGTOVTVSS |

The hinge regions of E165R-1, E165R-2, E165R-3, and E165R-4 sdAbs were highlighted in red

**Supplementary Table 3. Flow cytometry analysis data showing the binding of GUCY2C- and IL13Ra2-specific hcAbs to cell surface antigens.**

|  | 293T-GUCY2C | | 293T-IL13Ra2 | | 293T | |
| --- | --- | --- | --- | --- | --- | --- |
|  | % | GM | % | GM | % | GM |
| 5F9 | 83.30 | 17933 | 4.59 | 446 | 2.43 | 368 |
| 1608 | 77.60 | 11485 | 26.30 | 937 | 1.28 | 321 |
| V1 | 21.90 | 812 | 4.37 | 436 | 1.42 | 351 |
| V2 | 24.40 | 894 | 3.74 | 441 | 1.38 | 351 |
| V3 | 24.00 | 850 | 13.60 | 725 | 1.47 | 357 |
| V4 | 20.60 | 772 | 6.28 | 537 | 1.63 | 372 |
| 2D4 | 20.80 | 819 | 90.90 | 24381 | 3.05 | 374 |
| RV11-2 | 6.84 | 453 | 73.20 | 8834 | 1.17 | 321 |
| RV15 | 5.04 | 392 | 71.70 | 8394 | 0.92 | 315 |
| RV14 | 4.01 | 410 | 62.00 | 5040 | 0.83 | 304 |
| NC | 2.80 | 371 | 3.30 | 417 | 0.84 | 321 |

The % denotes the percentage of human-IgG-Fc^+^ cells and GM means Geometric Mean.

**Supplementary Table 4. Affinity analysis of the screened GUCY2C or IL13Ra2 specific sdAbs and hcAbs.**

|  | **KD (M)** | **kdis (1/s)** | **kon (1/Ms)** | **Full R^2^** |
| --- | --- | --- | --- | --- |
| GUCY2C-V1-M | 1.36 × 10^-7^ | 4.24 × 10^-3^ | 3.11 × 10^4^ | 0.9301 |
| GUCY2C-V2-M | 7.02 × 10^-5^ | 3.21 × 10^-2^ | 4.58 × 10^2^ | 0.9412 |
| GUCY2C-V3-M | 2.68 × 10^-6^ | 2.03 × 10^-2^ | 7.58 × 10^3^ | 0.9512 |
| GUCY2C-V4-M | 3.00 × 10^-7^ | 1.19 × 10^-2^ | 1.78 × 10^4^ | 0.9318 |
| GUCY2C-V1-B | 5.44 × 10^-8^ | 1.19 × 10^-3^ | 2.19 × 10^4^ | 0.9738 |
| GUCY2C-V2-B | 4.62 × 10^-9^ | 1.08 × 10^-3^ | 2.34 × 10^4^ | 0.9756 |
| GUCY2C-V3-B | 5.19 × 10^-8^ | 1.13 × 10^-3^ | 2.18 × 10^4^ | 0.9766 |
| GUCY2C-V4-B | 4.74 × 10^-9^ | 8.41 × 10^-4^ | 1.77 × 10^4^ | 0.9754 |
| IL13Ra2-2D4-M | 1.07 × 10^-9^ | 7.17 × 10^-4^ | 6.68 × 10^5^ | 0.9615 |
| IL13Ra2-RV11-2-M | 5.30 × 10^-8^ | 6.50 × 10^-4^ | 1.23 × 10^4^ | 0.9563 |
| IL13Ra2-RV15-M | 3.73 × 10^-8^ | 2.12 × 10^-3^ | 5.68 × 10^4^ | 0.9303 |
| IL13Ra2-RV14-M | 9.96 × 10^-8^ | 9.56 × 10^-3^ | 9.59 × 10^4^ | 0.9479 |
| IL13Ra2-2D4-B | 7.60 × 10^-8^ | 7.26 × 10^-4^ | 9.56 × 10^3^ | 0.9762 |
| IL13Ra2-RV11-2-B | 3.18 × 10^-8^ | 3.23 × 10^-4^ | 1.02 × 10^4^ | 0.9896 |
| IL13Ra2-RV15-B | 3.57 × 10^-8^ | 3.83 × 10^-4^ | 1.07 × 10^4^ | 0.9767 |
| IL13Ra2-RV14-B | 3.93 × 10^-8^ | 2.90 × 10^-4^ | 7.38 × 10^3^ | 0.9924 |

The dissociation constant (KD (M)), on-rates (kon (1/Ms)) and off-rates (kdis (1/s)) for monovalent sdAbs (M) or bivalent Fc-fused hcAbs (B) binding to GUCY2C or IL13Ra2.

**Supplementary** **Table 5.** **List of sgRNA sequences and primers for MDG1 mice genotyping.**

| **sgRNA sequence** |  |
| --- | --- |
| sgRNA1 | GACTCTCTGAGGACCAGAGA |
| sgRNA2 | GATCTGCATGTGCCCATTCC |
| sgRNA3 | AGTGGACAAGAAAATTGGTG |
| sgRNA4 | CTTGTGAACTTCTACTCCCC |
| **primers for genotyping** |  |
| 1F | GTTCATGCCCCTAGAGTTGG |
| 1R | TACCATTCTCCCTGGAGTGG |
| 2R | ATCTCTGCGACAGCTGGAAT |

**Supplementary Table 6.** **List of primers for RT-PCR and q-PCR.**

| **primers for RT-PCR** |  |  |
| --- | --- | --- |
| JH2 | CTACTGGGGCCAAGGCAC |  |
| IgG2c-CH3-R | CAGGTAAGAAGCCTGTGATC |  |
| **primers for q-PCR** |  |  |
| JH2 | CTACTGGGGCCAAGGCAC | *μ* |
| q-IgM-CH1-R | AGCCCATGGCCACCAGATTC |  |
| q-IgG2c-hinge-CH2-F | ATGCGCAGCTCCAGACCTCTTG | *γ2c* |
| q-IgG2c-CH2-R | GGCTCAGGGAGATCATGAG |  |
| JH2-3 | TACTGGGGCCAAGGCACCACTC | *α* |
| q-IgA-CH1-R | GGGTCACTTGACAGAGCTCGTG |  |
| Q-GAPDH-F | AGGTCGGTGTGAACGGATTTG | *gapdh* |
| Q-GAPDH-R | TGTAGACCATGTAGTTGAGGTCA |  |

**Supplementary Table 7.** **List of primers for NGS library construction of IgG2c and IgM.**

| **The first round** |  |
| --- | --- |
| MHV1 | ATGAAATGCAGCTGGGGCATSTTCTTC |
| MHV2 | ATGGGATGGAGCTRTATCATSYTCTT |
| MHV3 | ATGAAGWTGTGGTTAAACTGGGTTTTT |
| MHV4 | ATGRACTTTGGGYTCAGCTTGRTTT |
| MHV5 | ATGGGACTCCAGGCTTCAATTTAGTTTTCCTT |
| MHV6 | ATGGCTTGTCYTTRGSGCTRCTCTTCTGC |
| MHV7 | ATGGRATGGAGCKGGRGTCTTTMTCTT |
| MHV8 | ATGAGAGTGCTGATTCTTTTGTG |
| MHV9 | ATGGMTTGGGTGTGGAMCTTGCTTATTCCTG |
| MHV10 | ATGGGCAGACTTACCATTCTCATTCCTG |
| MHV11 | ATGGATTTTGGGCTGATTTTTTTTATTG |
| MHV12 | ATGATGGTGTTAAGTCCTTCTGTACC |
| IgG2c-CH2-R | TGGTCCACCCAAGAGGTCTG |
| IgM-CH2-R | GTTCTCGATGGTCACCGGATC |
| **The second round** |  |
| MHVF1 | GAGGTGAAGCTTCTCGAGTCTGG |
| MHVF2 | SAGGTSCAGCTGMAGGAGTCWGG |
| MHVF3 | GAGGTCCAGCTGCAACAATCTGG |
| MHVF4 | SAGGTYCARCTKCAGCAGYCTGG |
| MHVF5 | GARGTGAAGCTTGWGGAGTCTGG |
| WT-IgG2c-CH1-R | GGCCAGTGGATAGACCGATG |
| KO-IgG2c-CH2-R | TCCACCCAAGAGGTCTGGAG |
| WT-IgM-CH1-R | GACATTTGGGAAGGACTGAC |

**Supplementary Table 8.** **List of primers for NGS library construction of Ig*κ*.**

| **The first round** |  |
| --- | --- |
| IGKV1 | AGATTTTCAGCTTVCTGCTAATCWG |
| IGKV2 | TTGGSHTCTTGTTGCTCTKGTT |
| IGKV3 | AGAYYCAGGYCTTYGTATT |
| IGKV4 | ATGGARTCACAKACTCWGGTC |
| IGKV5 | ATGGWATCASAGRCCCAKGT |
| IGKV6 | TTGGACTTHTGCTTTTCTGGA |
| IGKV7 | GTTGCTGCTGCTSTGGSTTMCA |
| IGKV8 | ATGAAGTTTCCTTCTCAACTTC |
| IGKV9 | CTGTCCAGTTCCTGTTTCTG |
| IGKV10 | CTTCTKTTGYTCTGGATYCCT |
| IGKV11 | TCTCCTCAGYCTTCTTCTCCTC |
| IGKV12 | GGGTGTACCCACTCAGCTCCTG |
| IGKV13 | TSYTGATGYTCTGGCTYCA |
| IGKC-R | GACTGAGGCACCTCCAGATG |
| **The second round** |  |
| IGKVF1 | SAAAWTGTKCTCACCCAGTCTC |
| IGKVF2 | GACATTSTGMTRACYCAGTCTC |
| IGKVF3 | RACATTGTRATGACCCARWCTC |
| IGKVF4 | GACATTDTGHTGACMCARTC |
| IGKVF5 | GACATCCAGATGACHCARTCT |
| IGKVF6 | GAAACAACTGTGACCCAGTCTC |
| IGKVF7 | AATATCCAGGTGATCCAGTCAC |
| IGKVF8 | GATGTTGTGATGACBCARRCT |
| IGKVF9 | GAAATTGTGCTGACTGTGTCAG |
| IGKC-R2 | GATACAGTTGGTGCAGCATC |

**Supplementary Table 9.** **List of primers for phage display library construction.**

| **The second round** |  |
| --- | --- |
| MHVF1-*Sfi* Ⅰ-F | ATGCCATGACTGTggcccaggcggccGAGGTGAAGCTTCTCGAGTCTGG |
| MHVF2-*Sfi* Ⅰ-F | CATGCCATGACTGTggcccaggcggccSAGGTSCAGCTGMAGGAGTCWGG |
| MHVF3-*Sfi* Ⅰ-F | CATGCCATGACTGTggcccaggcggccGAGGTCCAGCTGCAACAATCTGG |
| MHVF4-*Sfi* Ⅰ-F | CATGCCATGACTGTggcccaggcggccSAGGTYCARCTKCAGCAGYCTGG |
| MHVF5-*Sfi* Ⅰ-F | CATGCCATGACTGTggcccaggcggccGARGTGAAGCTTGWGGAGTCTGG |
| IgG2c-CH2-*Sfi* Ⅰ-R | ACTCGCGGCCGGCCTGGCCTCCACCCAAGAGGTCTGGAG |
